# Uncommon trans-iliac bone faecal fistula in a leukaemic patient

**DOI:** 10.1259/bjrcr.20190056

**Published:** 2020-02-12

**Authors:** Lucilene Silva Ruiz Resende, Seizo Yamashita, Ligia Niéro-Melo

**Affiliations:** 1 Haematology Service of Internal Medicine Department, Botucatu Medical School, São Paulo State University - UNESP, Botucatu, Brazil; 2 Radiology Service of Tropical Diseases and Imaging Diagnosis Department, Botucatu Medical School, São Paulo State University - UNESP, Botucatu, Brazil

## Abstract

This is a rare case of an elderly woman diagnosed with acute myeloid leukaemia secondary to myelodysplastic syndrome who presented a spontaneous trans-iliac bone faecal fistula probably related to an incarcerated inguinal hernia and neutropaenia. As far as we know, this is the first described case of a trans-iliac bone faecal fistula.

## Case presentation

An 83-year-old white female was diagnosed with myelodysplastic syndrome (MDS). She had neglected an incarcerated right inguinal hernia for many years which was resistant to any attempt at manual reduction. In the following year, she presented recurrent episodes of abdominal pain, vomiting and fever, and was hospitalized several times for antibiotics due to a clinical diagnosis of neutropaenic enterocolitis. However, repeated abdominal ultrasonography never showed bowel wall thickening or perforation. When the MDS evolved into acute myeloid leukaemia (AML) the patient was admitted in poor condition, presenting fever, abdominal pain, vomiting, and a 10 cm diameter inflammatory swelling in her right gluteal region which was thought to be a cold abscess due to her neutropaenic status. A general surgeon was called to perform surgical drainage of the lesion.

## Investigation

**Figure 1.**
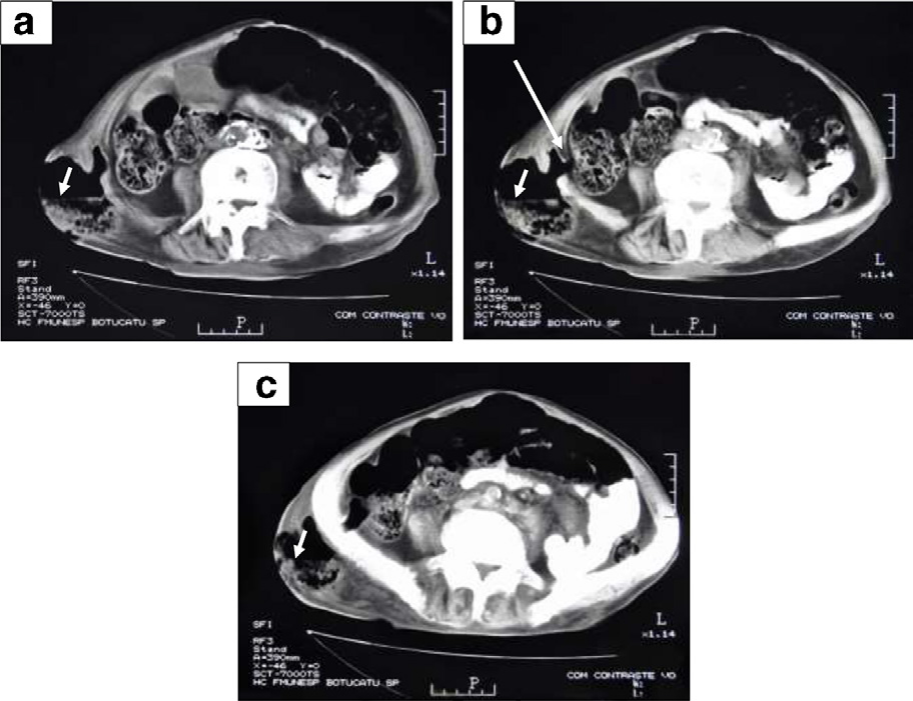
﻿Figure 1- a, b, c: CT scan with oral contrast: axial sections showing a faecal fistula crossing through the right iliac bone in its way from the colon to a gluteous reservoir. Complete fistula track can be seen only on Figure 1b (big arrow). Other pictures correspond to images taken respectively above (Figure 1a) and below (Figure 1c) that one. An air-feces level can be seen in the gluteous reservoir (small arrows).

Peripheral blood showed a haemoglobin concentration of 6.8 g/dL, platelet count of 69 × 10^9^/L, and leukocyte count of 1.7 × 10^9^/L (48% blasts, 10% neutrophils, 34% lymphocytes, 8% monocytes). The attempt to surgically drain the supposed abscess resulted in liquid faeces discharge through the cutaneous incision. Only then was the patient sent for CT which revealed a faecal fistula (Figure 1).

## Diferential diagnosis

The gluteal swelling was initially thought to be a cold abscess as a result of the patient’s neutropaenic status related to AML. Neutropaenic patients are immunosuppressed and lack the ability to mount an inflammatory response, and may not present the typical signs and symptoms of infection.

## Treatment

She was admitted to hospital and cefepime, vancomycin, plus clindamycin were empirically initiated. After diagnosis of the trans-iliac bone faecal fistula by CT, a surgical gastroenterology evaluation was requested. The patient was managed conservatively due to her advanced age, poor condition, underlying haematological disease, and neutropaenia. Only a colostomy bag was placed on the lesion.

## Outcome and follow-up

Only palliative care was maintained. No chemotherapy was administered. Physiological intestinal transit and faecal fistula drainage persisted until death, which occurred 15 days later due to sepsis. Necropsy was not performed.

## Discussion

Spontaneous faecal fistulas are usually associated with previous abdominal surgery or trauma, inflammatory or infectious bowel diseases, diverticulitis, necrotizing pancreatitis, hernia mesh erosion, radiation, and several invasive medical procedures, among others.^1–3^ Inguinal hernia can cause spontaneous faecal fistula^4,5^ as a rare complication.^5,6^ This kind of fistula seems to be very uncommon in neutropaenic patients although it has been previously described in a patient specific diagnosed with cyclic neutropaenia.^7^ A neutropaenic status, however, can predispose patients to neutropaenic enterocolitis which affects the colon, mainly the cecum, and/or the small bowel.^8,9^ Oedema, necrosis, haemorrhage and ulceration usually occur, and evolution to bowel perforation is also possible.^9^

The patient presented spontaneous trans-iliac bone faecal fistula related to a neglected and chronically incarcerated inguinal hernia. Her neutropaenic status, which worsened when MDS transformed into AML, could also have played a role in the physiopathology of the fistula formation. She just presented findings which were clinically thought to be recurrent episodes of neutropaenic enterocolitis^8,9^ but repeated abdominal ultrasonography never confirmed this. The iliac bone was probably no obstacle to fistulization because of patient osteoporosis secondary to advanced age and bed confinement, added to possible bone weakness due to the underlying bone marrow disease.

We could not find any cases of faecal fistula due to an underlying disease or condition which have taken a trans-iliac bone route from the intestine to the site of faecal discharge. This case report is being published in view of its singularity.

## Learning points

1. Patients suffering from immunosuppressive haematological diseases can present rare and unexpected complications.
2. Their complications can be mostly atypical or subclinical, evolving differently from those of immunocompetent patients.
3. A combination of findings including old age, bone frailty, chronic neutropaenic status and chronic partial obstacle to normal faecal flow by chronically incarcerated inguinal hernia may have contributed to this rare trans-iliac bone faecal fistula.

## References

[1] Losanoff J, Richman B, Jones J Entero-colocutaneous fistula: a late consequence of polypropylene mesh abdominal wall repair: case report and review of the literature. Hernia 2002; 6: 144–7. doi: 10.1007/s10029-002-0067-z 12209305

[2] Hollington P, Mawdsley J, Lim W, Gabe SM, Forbes A, Windsor AJ An 11-year experience of enterocutaneous fistula. Br J Surg 2004; 91: 1646–51. doi: 10.1002/bjs.4788 15505866

[3] Camprodon RAM, Jacob S, George ML, Karani JB, Leather AJM Colocutaneous fistula complicating therapeutic mesenteric embolisation. Ann R Coll Surg Engl 2007; 89: 1–3. doi: 10.1308/147870807X188443 17688707 PMC2048622

[4] Rao PL, Mitra SK, Pathak IC Fecal fistula developing in inguinal hernia. Indian J Pediatr 1980; 47: 253–5. doi: 10.1007/BF02758204 7239629

[5] Samad A, Sheikh GM Spontaneous faecal fistula: a rare presentation of inguinal hernia. J Ayub Med Col Abbottabad 2005; 17: 1–3. 16599044

[6] Koshariya M, Naik S, Rai A Incarcerated inguinal hernia presenting as spontaneous scrotal fecal fistula. Hernia 2006; 10: 434–5. doi: 10.1007/s10029-006-0119-x 16897640

[7] Li D-Y, Scheimann AO, Songer JG, Person RE, Horwitz M, Resar L, et al Enteritis necroticans with recurrent enterocutaneous fistulae caused by Clostridium perfringens in a child with cyclic neutropenia. J Pediatr Gastroenterol Nutr 2004; 38: 213–5. 14734887 10.1097/00005176-200402000-00021

[8] Gorschlüter M, Mey U, Strehl J, Ziske C, Schepke M, Schmidt-Wolf IGH, et al Neutropenic enterocolitis in adults: systematic analysis of evidence quality. Eur J Haematol 2005; 75: 1–13. doi: 10.1111/j.1600-0609.2005.00442.x 15946304

[9] Davila ML Neutropenic enterocolitis: current issues in diagnosis and management. Curr Infect Dis Rep 2007; 9: 116–20. doi: 10.1007/s11908-007-0006-3 17324348

